# Head and Neck Tumor Segmentation for MRI-Guided Radiation Therapy Using Pre-trained STU-Net Models

**DOI:** 10.1007/978-3-031-83274-1_4

**Published:** 2025-03-03

**Authors:** Zihao Wang, Mengye Lyu

**Affiliations:** 1 College of Health Science and Environmental Engineering, Shenzhen Technology University, Shenzhen, China; 2 College of Applied Sciences, Shenzhen University, Shenzhen, China

**Keywords:** Medical image segmentation, STU-Net, nnU-Net, tumor segmentation, MRI-guided radiation therapy

## Abstract

Accurate segmentation of tumors in MRI-guided radiation therapy (RT) is crucial for effective treatment planning, particularly for complex malignancies such as head and neck cancer (HNC). This study presents a comparative analysis between two state-of-the-art deep learning models, nnU-Net v2 and STU-Net, for automatic tumor segmentation in pre-RT MRI images. While both models are designed for medical image segmentation, STU-Net introduces critical improvements in scalability and transferability, with parameter sizes ranging from 14 million to 1.4 billion. Leveraging large-scale pre-training on datasets such as TotalSegmentator, STU-Net captures complex and variable tumor structures more effectively. We modified the default nnU-Net v2 by adding additional convolutional layers to both the encoder and decoder, improving its performance for MRI data. Based on our experimental results, STU-Net demonstrated better performance than nnU-Net v2 in the head and neck tumor segmentation challenge. These findings suggest that integrating advanced models like STU-Net into clinical work-flows could remarkably enhance the precision of RT planning, potentially improving patient outcomes. Ultimately, the performance of the fine-tuned STU-Net-B model submitted for the final evaluation phase of Task 1 in this challenge achieved a DSCagg-GTVp of 0.76, a DSCagg-GTVn of 0.85, and an overall DSCagg-mean score of 0.81, securing ninth place in the Task 1 rankings. The described solution is by team SZTU-SingularMatrix for Head and Neck Tumor Segmentation for MR-Guided Applications (HNTS-MRG) 2024 challenge. Link to the trained model weights: https://github.com/Duskwang/Weight/releases.

## 1 Introduction

Radiation therapy (RT) plays a pivotal role in the management of various malignancies, particularly head and neck cancer (HNC) [1, 2]. The introduction of MRI-guided RT planning has gained substantial interest in recent years, largely due to the superior soft tissue contrast that MRI provides compared to traditional CT-based approaches [3]. MRI not only enhances anatomical visualization but also enables functional imaging through advanced multiparametric sequences, such as diffusion-weighted imaging. These capabilities are further leveraged by MRI-Linac systems, which allow for daily adaptive RT through real-time intra-therapy imaging and adjustments, optimizing tumor targeting while minimizing collateral damage [4–6]. These technological advancements have the potential to transform clinical practices for HNC treatment.

However, the integration of MRI into RT planning comes with challenges, particularly in the context of image segmentation. Accurate tumor segmentation is crucial for effective RT planning, yet the manual segmentation of MRI data is time-consuming and complex, especially given the intricate anatomy of HNC [7, 8]. The high resolution and detailed contrast of MRI, while advantageous, result in large datasets that are labor-intensive for clinicians to process. Moreover, the variability in MRI appearances and the need for consistent, reproducible segmentation further complicate the task.

To address these challenges, artificial intelligence (AI) methods, particularly deep learning, have shown great promise in automating the segmentation of HNC tumors [9]. A notable advancement in this field is the development of STU-Net, a scalable and transferable model designed for medical image segmentation. STU-Net builds upon the popular nnU-Net framework but introduces key improvements to enhance scalability and transferability. It can be scaled to sizes ranging from 14 million to 1.4 billion parameters, making it the largest medical image segmentation model to date [10]. By leveraging large-scale pre-training on datasets, STU-Net is capable of generalizing across various medical imaging tasks, including those involving CT, MRI, and PET scans. Its ability to scale in both depth and width allows it to achieve superior performance, especially in handling the complex and variable structures encountered in HNC segmentation.

In this paper, we apply the STU-Net framework to the segmentation of HNC tumors in the context of MRI-guided adaptive RT and compare it with nnU-Net based models. Overall, the results highlight the clear advantage of STU-Net for precise tumor segmentation in this context.

## 2 Methods

### 2.1 Model Architecture

In this study, we used two state-of-the-art deep learning frameworks for fully automatic tumor volume segmentation on pre-RT MRI: nnU-Net v2 and STU-Net. Both architectures are designed for medical image segmentation, but they offer distinct advantages in terms of flexibility, scalability, and performance.

#### 1. nnU-Net V2:

The nnU-Net v2 [11] framework is an advanced version of the nnU-Net, featuring improved self-configuring capabilities and enhanced adaptability for medical image segmentation tasks. It builds upon the original U-Net architecture with refined self-configuration that includes optimized net-work depth, input patch size, and training parameters based on the input data’s characteristics [11]. nnU-Net v2 incorporates advanced training strategies, flexible data preprocessing, and improved upsampling methods, which collectively enhance its performance and scalability. For this study, we utilized the default 3D full-resolution nnU-Net v2 configuration, which has shown exceptional robustness in medical image segmentation tasks, including tumor delineation in MRI images [12].

The nnU-Net v2 framework was trained using the HNC dataset in a supervised manner. The model’s advanced self-adaptation capabilities without requiring manual tuning make it a solid baseline for comparison. However, given the complexity and variability of HNC tumors in MRI images, we identified opportunities for further improvement with a more scalable and context-aware approach.

#### 2. STU-Net:

STU-Net [10] is developed from the nnU-Net framework to address the challenges of scalability and transferability in large-scale medical image segmentation models. It improves upon nnU-Net by refining convolutional blocks, replacing upsampling methods, and fixing hyperparameters to enhance model scalability and transferability [10]. Additionally, by pre-training on the large-scale dataset, STU-Net demonstrates exceptional performance across various medical image segmentation tasks. By scaling both the width and depth of the network, STU-Net can capture both fine-grained local details and long-range dependencies [10], which are crucial in segmenting complex tumor structures like those found in HNC.

STU-Net’s design allows for larger models, with parameter sizes ranging from 14 million to 1.4 billion. In this study, we trained STU-Net with official pre-trained weights of different sizes, followed by fine-tuning on the HNC MRI data. This transfer learning approach allowed STU-Net to leverage global structural information from the pre-training phase, while fine-tuning on the HNC-specific data further refined the model for better segmentation accuracy.

### 2.2 Training Strategies

We conducted a series of experiments to evaluate the impact of different training strategies on segmentation performance:

#### 1. Standard nnU-Net V2:

In this study, we utilized the publicly available nnU-Net v2 framework, which was installed directly from its official GitHub repository. The segmentation process was carried out using the default configuration provided by the nnU-Net v2 framework, which automatically determines optimal network depth, input patch size, and training parameters based on the characteristics of the input data. No manual tuning or customization of hyperparameters was applied, ensuring that the results reflect the baseline performance of nnU-Net v2 for the given dataset.

#### 2. nnU-Net V2*:

We modified the default nnU-Net v2 configuration by increasing the number of convolutional layers in each stage of the encoder from 2 to 3. This adjustment aims to enhance the feature extraction capabilities at each resolution level, potentially improving the model’s ability to capture complex tumor structures in MRI images of the head and neck. The added convolutional layers may help the model better delineate tumor boundaries, potentially improving segmentation accuracy, particularly for more challenging anatomical regions.

#### 3. nnU-Net V2^**^:

Based on nnU-Net v2*, we increased the number of convolutional layers in each stage of the decoder from 2 to 3. This further adjustment enhances the model’s feature extraction and reconstruction capabilities, enabling it to better capture and restore complex details in MRI images of head and neck cancer, ultimately improving segmentation accuracy.

#### 4. STU-Net-S:

By simultaneously scaling the encoder and decoder structures in STU-Net, and scaling the depth and width at each resolution stage by the same ratio, different scales of STU-Net can be obtained [10]. We first used a small-scale model referred to as STU-Net-S, which has 14.60M parameters [10]. This design aims to enhance performance at a relatively lower computational cost, particularly in capturing finer details in head and neck tumor images.

#### 5. STU-Net-B:

Expanding the width of STU-Net-S results in the STU-Net-B model, which has 58.26M parameters [10]. STU-Net family also includes two even larger scale models (STU-Net-L and STU-Net-H), which were not investigated in this study considering the high hardware requirement. The purpose of expanding the width is to improve the model’s feature extraction capabilities, potentially enabling it to better capture complex tumor features.

#### 6. STU-Net-B*:

To determine whether the improved segmentation performance is due to the STU-Net architecture itself or the effect of pre-training, we conducted experiments without loading the pre-trained weights. This allowed us to assess the contribution of each factor. We denote the STU-Net-B model without pre-trained weights as STU-Net-B*.

### 2.3 Evaluation Metrics

To evaluate the segmentation results for challenge Task 1 (pre-RT segmentation), we used the aggregated Dice Similarity Coefficient (DSCagg) metric provided officially. DSCagg is calculated separately for GTVp (DSCagg-GTVp) and GTVn (DSCagg-GTVn), with the final segmentation performance based on the average of these two values (DSCagg-mean). Additionally, we also use the traditional Dice Similarity Coefficient (DSC) as an evaluation metric [13]. The expected labels for the predicted masks are as follows: 1 for GTVp, 2 for GTVn, and 0 for the background.

### 2.4 Datasets

The official head and neck tumor dataset consists of 150 cases. For this study, we selected Task 1 and exclusively used pre-RT data for training. The dataset was randomly split, with 30 cases designated as the internal validation set, while the remaining 120 cases were used for model training.

### 2.5 Implementation Details

The experiments were implemented using Python and the PyTorch deep learning library. Training was conducted on a GPU Server. The training protocols are presented in Table 1. The hardware configuration and development environments are presented in Table 2.

## 3. Results

The comparative performance analysis between nnU-Net v2 and STU-Net revealed clear advantages for STU-Net in the segmentation of head and neck cancer tumors. Considering the limitations of computational resources and the prolonged training time due to the use of a less powerful GPU, this study conducted a single training-validation split for model training and evaluation. While this approach may not fully capture the variability across different data partitions, it was adopted as a practical compromise to ensure the feasibility of the experiments within the available resource constraints. Table 3 presents the quantitative results of all evaluation metrics derived from the model training conducted using a single training-validation split approach.

nnU-Net v2 achieved an average DSCagg-mean of 0.77, with DSCagg-GTVp at 0.75 and DSCagg-GTVn at 0.78. nnU-Net v2* improved slightly with a DSCagg-mean of 0.74, reflecting better feature extraction capabilities in the encoder. nnU-Net v2^**^ with both the encoder and decoder enhanced showed a modest improvement in GTVn segmentation with a DSCagg-mean of 0.75.

STU-Net-S outperformed the nnU-Net variants, achieving a DSCagg-mean of 0.82, with remarkable improvements in both GTVp (0.79) and GTVn (0.84). STU-Net-B, the base version of STU-Net, performed even better, with a DSCagg-mean of 0.83, showcasing its ability to capture more complex features in HNC MRI data.

When training without pre-trained weights, STU-Net-B* achieved a DSCagg-mean of 0.81. This indicates that although STU-Net (without pre-trained weights) still performed better than nnU-Net v2, it did not reach the performance of its pre-trained counterparts (STU-Net-S and STU-Net-B). This suggests that the pre-training phase substantially contributes to the model’s superior performance, particularly in the complex segmentation task of head and neck cancer.

In Table 3, “Test” refers to the performance of the trained STU-Net-B model submitted for the final evaluation phase of Task 1 in this challenge, achieving a DSCagg-mean of 0.81.

These results indicate that STU-Net demonstrates promising segmentation performance, particularly in delineating the intricate boundaries of HNC tumors on the validation set, though further validation on large-scale independent datasets is necessary. The scalability of STU-Net allowed it to effectively model both local and global structures, contributing to its higher Dice scores. Further-more, the pre-training on the large-scale dataset provided a solid foundation for transfer learning, allowing the model to generalize better across different imaging tasks.

Additionally, the average training time per epoch varied across strategies: 378.72 s for the default nnU-Net v2, 530.9 s for nnU-Net v2^**^, 418 s for STU-Net-B, and 198 s for STU-Net-S. These differences reflect the variations in model complexity and parameter counts, which influence the computational cost and training duration.

**Fig. 1.**
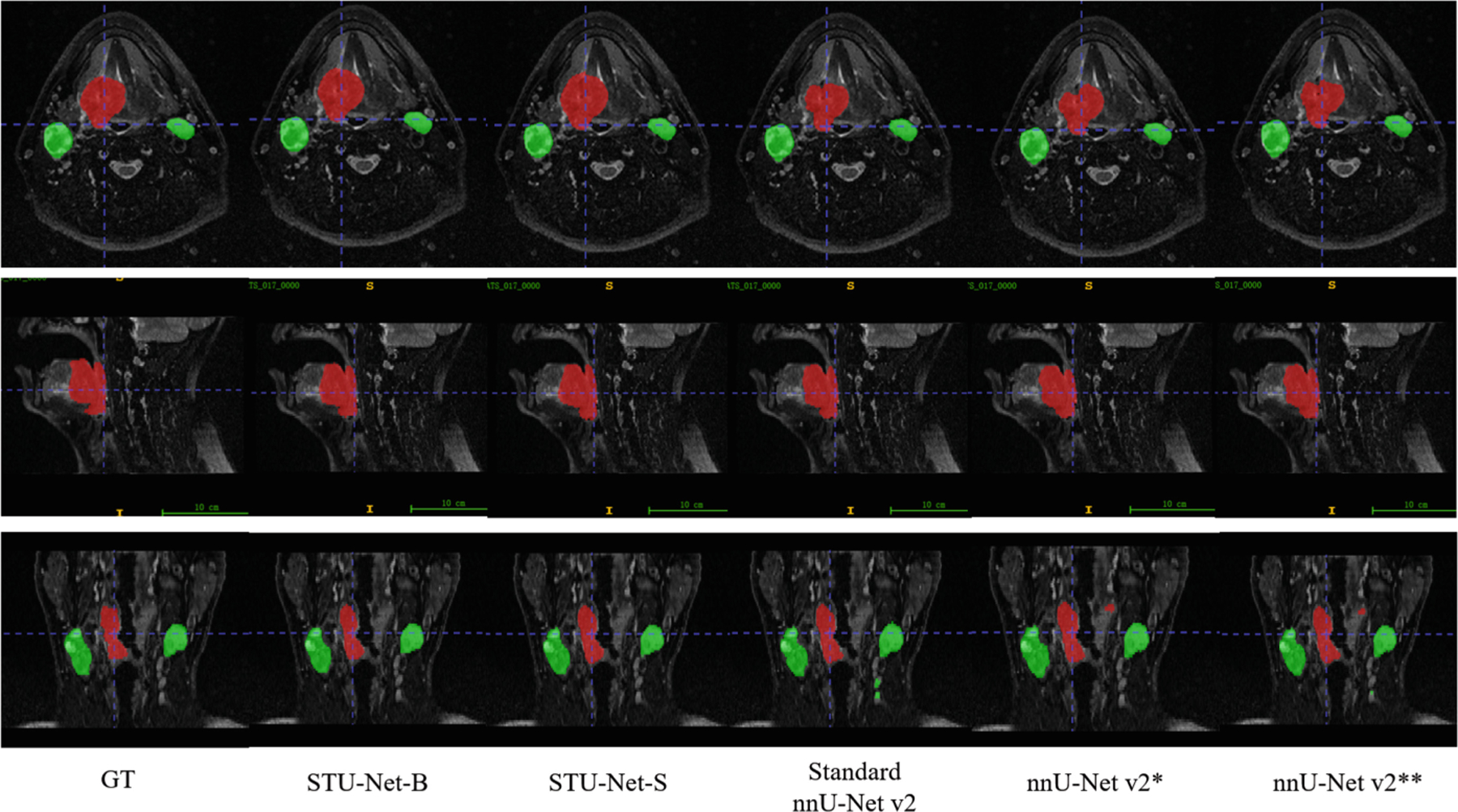
Qualitative visualization of different models.

In qualitative analyses (Fig. 1), STU-Net demonstrated a more precise segmentation of tumor boundaries compared to the nnU-Net variants, particularly in cases with high variability in tumor shape and size. This suggests that the architectural enhancements in STU-Net, combined with its ability to scale in depth and width, offer substantial advantages in handling the complexities of MRI-guided RT planning for HNC.

Overall, the results suggest that STU-Net, with its improved scalability and transferability, shows potential as a more accurate and reliable approach for tumor segmentation in MRI-guided RT compared to nnU-Net v2, but further verification is required.

## 4 Discussion

In this study, we explored the performance of two state-of-the-art deep learning models, nnU-Net v2 and STU-Net, for automatic tumor segmentation in pre-RT MRI of head and neck cancer (HNC). While both frameworks are designed to handle medical image segmentation, our findings indicate that STU-Net consistently outperformed nnU-Net v2 in this context, particularly when segmenting the complex and heterogeneous structures characteristic of HNC tumors.

The superior performance of STU-Net can be attributed to several key factors. First, the pre-training of STU-Net on the large-scale dataset provides substantial advantage over randomly initialized models. This pre-training allowed the model to leverage global structural information across multiple medical imaging modalities, enhancing its transferability and fine-tuning capacity on the HNC-specific MRI data. In comparison, the nnU-Net v2, while effective as a baseline model, lacked the same level of transfer learning benefits.

To investigate the contribution of pre-training, we trained a version of STU-Net without pre-trained weights, denoted as STU-Net-B*. While STU-Net-B* performed better than nnU-Net v2, its performance did not reach the levels of the pre-trained STU-Net models. This clearly indicates that pre-training plays a crucial role in enhancing STU-Net’s ability to capture complex tumor features and produce more accurate segmentation results.

Additionally, the ability to scale both the depth and width of the network allowed STU-Net to effectively capture fine-grained details while also modeling long-range dependencies, which are crucial for delineating intricate tumor boundaries. In contrast, nnU-Net v2, though adaptable and self-configuring to certain degrees, was more constrained in its ability to handle such complexities due to its default architecture. This limitation became evident when dealing with the high variability in tumor morphology and tissue contrast present in MRI-guided RT data.

Another aspect potentially contributing to STU-Net’s superior performance is the incorporation of transformer modules, which can improve global context awareness [14]. This suggests that for complex tumor segmentation tasks, models that incorporate advanced context-aware mechanisms like Transformers may be better suited than traditional fully convolutional architectures.

Despite the promising results achieved with STU-Net, there are still limitations to consider. One notable challenge is the substantial computational cost associated with training large-scale models like STU-Net, particularly when dealing with 3D high-resolution MRI data. While STU-Net offers scalability, training the largest model configurations may require costly hardware, which could limit its accessibility in clinical settings with limited resources. However, the scalability feature also allows for adjustments to be made based on available computational power, making STU-Net a versatile option for a range of applications.

In the context of Task 2, which involves predicting tumor segmentation on unseen mid-RT images, STU-Net’s ability to model long-range dependencies and adapt to variable anatomical structures offers great potential. By leveraging its transfer learning capabilities and advanced architectural components, STU-Net could be well-suited for addressing the unique challenges posed by mid-RT segmentation. Future research should investigate the performance of STU-Net on this task, as well as its ability to generalize to other imaging modalities such as PET-CT or ultrasound, to validate its broader applicability.

In summary, the results of this study demonstrate that STU-Net provides a more effective solution for the segmentation of HNC tumors in MRI-guided RT planning compared to widely used nnU-Net v2 models. These findings suggest that integrating large-scale models like STU-Net into clinical workflows could improve the accuracy and efficiency of radiation therapy planning, potentially leading to better patient outcomes.

## Figures and Tables

**Table 1.** Training protocols.

Network initialization	normal initialization
Batch size	2
Patch size	48 *×* 225 *×* 225
Total epochs	1000
Optimizer	SGD with nesterov momentum (μ = 0.99)
Weight decay	3e-5
Loss function	dice and cross entrop loss
Initial learning rate (lr)	0.001

**Table 2.** Development environments and requirements.

System	Ubuntu 22.04
CPU	Intel(R) Xeon(R) CPU E5–2680 v4
RAM	128G
GPU (number and type)	P40
CUDA version	12.1
Programming language	Python 3.10.10
Deep learning framework	torch 2.1.2, torchvision 0.16.2

**Table 3.** Segmentation metrics by different methods.

	Class 1 (GTVp)		Class 2 (GTVn)		DSCagg-mean
	DSC	DSCagg-GTVp	DSC	DSCagg-GTVn	
Standard nnU-Net v2	0.56 *±* 0.34	0.75	0.60 *±* 0.35	0.78	0.77
nnU-Net v2*	0.57 *±* 0.32	0.73	0.57 *±* 0.35	0.75	0.74
nnU-Net v2**	0.55 *±* 0.34	0.73	0.66 *±* 0.31	0.77	0.75
STU-Net-B*	0.63 *±* 0.33	0.79	0.61 *±* 0.37	0.83	0.81
STU-Net-S	0.64 *±* 0.31	0.79	0.66 *±* 0.34	0.84	0.82
STU-Net-B	**0.66** *±* **0.30**	**0.81**	**0.72** *±* **0.27**	**0.84**	**0.83**
Test	*\*	0.76	*\*	0.85	0.81
